# Recollections of a Rebel Surgeon and Other Sketches, or in the Doctor’s Sappy Days

**Published:** 1900-05

**Authors:** 


RECOLLECTIONS OF A REBEL SURGEON AND OTHER SKETCHES, OR IN THE DOCTOR'S SAPPY DAYS. By F. E. DANIEL, M. D. Illustrated. Austin, Texas: Von Boeckmann, Schultze & Co. 1899.

To paraphase Pope, "A little humor is a dangerous thing." The printed page is a cruel test for many spoken words. To nothing is it more fatal than to humor, unless the wit that it preserves be of that high order that marks the work of genius. The accent, attitude, and gesture of the *raconteur*, on which so much of the force of the story depends, are all lost in type. And the pathetic suffers no less than the humorous. M. J. F.

